# Long non-coding RNA (LncRNA) HOTAIR regulates BMP9-induced osteogenic differentiation by targeting the proliferation of mesenchymal stem cells (MSCs)

**DOI:** 10.18632/aging.202384

**Published:** 2021-01-10

**Authors:** Ruidong Li, Wenwen Zhang, Zhengjian Yan, Wei Liu, Jiaming Fan, Yixiao Feng, Zongyue Zeng, Daigui Cao, Rex C. Haydon, Hue H. Luu, Zhong-Liang Deng, Tong-Chuan He, Yulong Zou

**Affiliations:** 1 Department of Orthopaedic Surgery, The Second Affiliated Hospital of Chongqing Medical University, Chongqing 400010, China; 2 Molecular Oncology Laboratory, Department of Orthopaedic Surgery and Rehabilitation Medicine, The University of Chicago Medical Center, Chicago, IL 60637, USA; 3 Department of Obstetrics and Gynecology, The Affiliated University-Town Hospital of Chongqing Medical University, Chongqing 401331, China; 4 Department of Orthopaedic Surgery, The First Affiliated Hospital of Chongqing Medical University, Chongqing 400016, China; 5 Department of Breast Surgery, The First Affiliated Hospital of Chongqing Medical University, Chongqing 400016, China; 6 Ministry of Education Key Laboratory of Diagnostic Medicine, Chongqing Medical University, Chongqing 400016, China; 7 Department of Orthopaedic Surgery, Chongqing General Hospital, Chongqing 400021, China

**Keywords:** long non-coding RNA, HOTAIR, BMP9, osteogenic differentiation, mesenchymal stem cell

## Abstract

Long non-coding RNAs are important regulators of biological processes, but their roles in the osteogenic differentiation of mesenchymal stem cells (MSCs) remain unclear. Here we investigated the role of murine HOX transcript antisense RNA (mHotair) in BMP9-induced osteogenic differentiation of MSCs using immortalized mouse adipose-derived cells (iMADs). Touchdown quantitative polymerase chain reaction analysis found increased mHotair expression in bones in comparison with most other tissues. Moreover, the level of mHotair in femurs peaked at the age of week-4, a period of fast skeleton development. BMP9 could induce earlier peak expression of mHotair during *in vitro* iMAD osteogenesis. Silencing mHotair diminished BMP9-induced ALP activity, matrix mineralization, and expression of osteogenic, chondrogenic and adipogenic markers. Cell implantation experiments further confirmed that knockdown of mHotair attenuated BMP9-induced ectopic bone formation and mineralization of iMADs, leading to more undifferentiated cells. Crystal violet staining and cell cycle analysis revealed that silencing of mHotair promoted the proliferation of iMAD cells regardless of BMP9 induction. Moreover, ectopic bone masses developed from mHotair-knockdown iMAD cells exhibited higher expression of PCNA than the control group. Taken together, our results demonstrated that murine mHotair is an important regulator of BMP9-induced MSC osteogenesis by targeting cell cycle and proliferation.

## INTRODUCTION

Fracture non-union is a clinically important condition in patients recovering from bone fracture, which requires urgent care to stimulate bone regeneration [1, 2]. Bone tissue engineering (BTE) requires three key components, including scaffolds, osteoprogenitors and osteogenic factors [3–6]. Significant progress has been made in identifying MSCs (mesenchymal stem cells) from different sources (embryo, bone marrow, fat tissue, etc.) and applying them as osteoprogenitors [3, 7, 8]. Meanwhile, various scaffolds have also been created to sustain cell proliferation and differentiation [9–11]. Despite these accomplishments, the goal of developing clinically safe and effective BTE methods has been hampered by insufficient understanding of the molecular mechanisms that govern osteogenesis. In the past decades, many osteogenic factors have been discovered, with BMPs (bone morphogenic proteins), Wnt and Notch signals considered among the most important [12–16]. We have previously demonstrated that BMP-9 is one of the most potent of the BMP family in inducing MSC osteogenesis [12, 17]. However, the underlying mechanisms responsible for its bone-stimulating activities in MSCs have not be satisfactorily deciphered.

LncRNAs are long non-coding RNAs that have emerged to be widely present across various species, with more than 25,000 of them having been identified in human [18]. LncRNAs are expressed in a cell-type and tissue-specific manner [19], and can be classified according to their relative location to protein-coding genes into five categories (sense, anti-sense, bidirectional, intronic and intergenic) [20, 21].

The HOX transcript antisense RNA (HOTAIR) [22], transcribed from the non-coding strand (i.e. antisense) of the HOX-C cluster in Chromosome 12, forms a chromatin-modifying complex with Polycomb Repressive Complex 2 (PRC2) and LSD1, and is delivered to chromosome 2 to induce histone H3 lysine-27 trimethylation (H3K27me3) of the HOX-D locus, leading to transcriptional repression of the downstream genes. Since its discovery, HOTAIR has been implicated in a variety of cellular, developmental and pathological processes [22–26]; however, literature report of its role in bone development and MSC osteogenesis is rather limited. Li et al. previously demonstrated that knockout of mHotair resulted in transformation of the spine and malformation of metacarpal-carpal bones in mice, thereby linking the lncRNA to bone development and osteogenic differentiation of MSCs [27]. Since BMP9 is one of the most osteogenic inducers, we were interested in probing the role of mHotair in BMP9-dependent MSC osteogenesis.

In this study, we investigated of mHotair in cellular experiments and in mice. Our experimental data indicated that mHotair is widely distributed across different tissues but mostly expressed in bones. The femoral level of mHotair peaked at week 4 postnatal in mice and coincided with fast skeleton development. Importantly, BMP9 could expedite peak mHotair expression during the osteogenic differentiation of MSCs. Silencing of mHotair was found to diminish BMP9-induced ALP activity, matrix mineralization, as well as expression of osteogenic, chondrogenic and adipogenic markers in iMAD cells. Consistently, cell implantation experiments confirmed that knockdown of mHotair attenuated BMP9-induced ectopic bone formation and resulted in more undifferentiated, fast-proliferating cells. Taken together, these results demonstrated that mHotair could play important roles in BMP9-induced MSC osteogenesis by regulating both cell differentiation and proliferation.

## RESULTS

### Expression of mHotair in mice tissues and cultured iMAD cells

**Figure 1:**
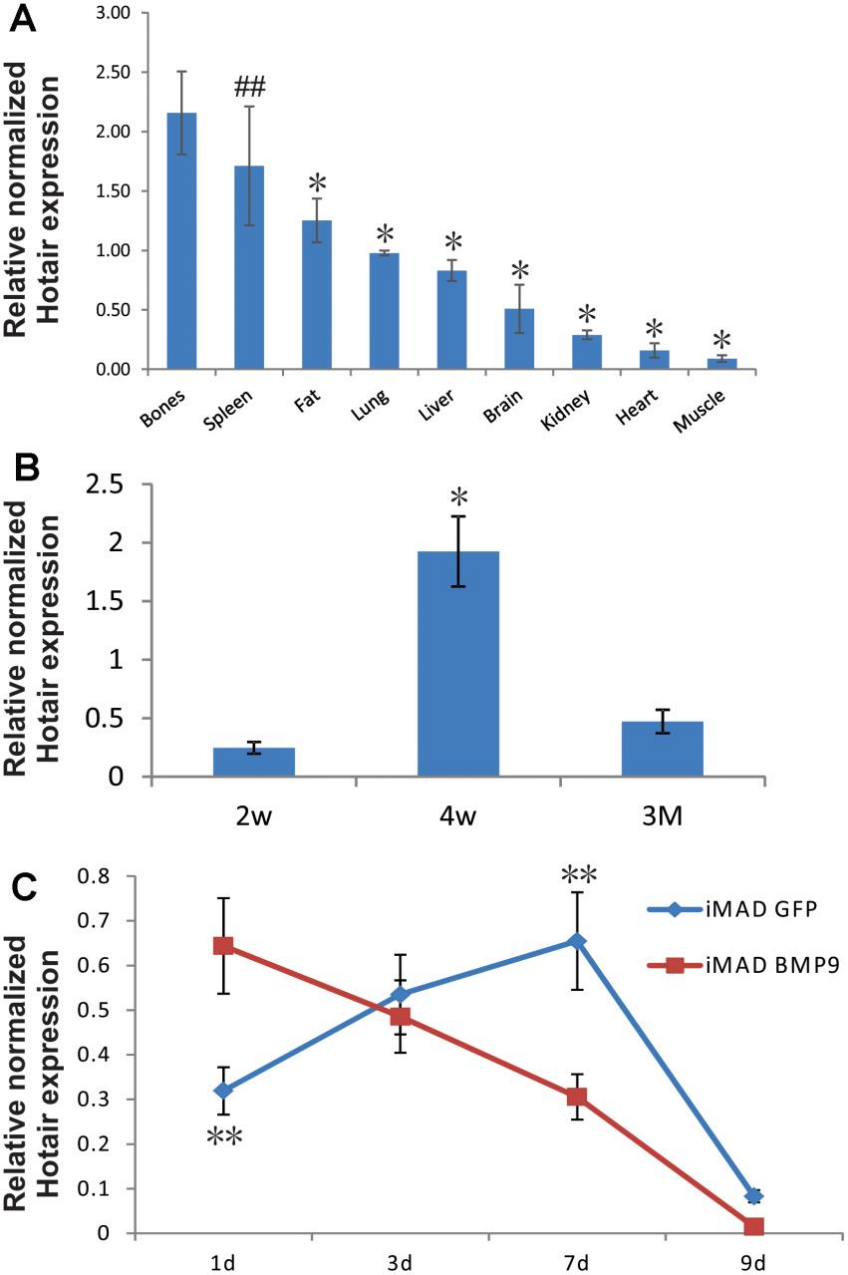
**Endogenous expression of HOTAIR in different tissues of mice and in MSCs undergoing osteogenic differentiation.** (**A**) Bones exhibit higher expression of HOTAIR than most other tissues. Total RNA from different tissues of 4-week-old male CD1 mice (N=5) was extracted and subjected to reverse transcription followed by TqPCR analysis. Reactions were done in triplicate. “##” p > 0.05, bones vs spleen, “*” p < 0.05, bones vs other tissues/organs. (**B**) Expression of HOTAIR in femurs was peaked at 4-week, a fast growing period for mice skeleton. Total RNA from different ages of CD1 mice femurs (N=5) was extracted and subjected to reverse transcription followed by TqPCR analysis. Reactions were done in triplicate. “*” p < 0.05, 4-week vs 2-week/3-month. (**C**) Endogenous expression of HOTAIR in iMADs is up-regulated during osteogenic differentiation and quickly dropped in the late stage, and BMP9 could potentially bring forward the peak of HOTAIR expression. Subconfluent iMAD cells were infected with Ad-GFP or Ad-BMP9. Total RNA was isolated at 48 h and subjected to TqPCR analysis using gene-specific primers for mouse HOTAIR. Gapdh was used as a reference gene. Reactions were done in triplicate. “**” p < 0.05, iMAD GFP group vs iMAD BMP9 group.

We first analyzed the expression of mHotair in different murine tissues. Total RNA was prepared from nine types of tissues in 4-week-old male CD1 mice (N = 5), and subjected to reverse transcription and then TqPCR analysis. The results showed that mHotair levels were generally greater in bones (TV, PB, femurs, and ribs) than in any other tissues except for spleen (Figure 1A). We also quantified mHotair in the femurs of adolescent mice at three different ages and found that its expression peaked at week 4 after birth (Figure 1B), a period characterized by rapid skeletal development. In cell-based assays, the transcriptional level of mHotair in GFP-treated iMAD cells exhibited an increase during the first seven days and then decreased dramatically from day 9. In comparison, mHotair expression in BMP9-induced iMAD cells was already at its highest level at day 1 and was approximately twice as much as that in the GFP group, but rapidly declined afterwards (Figure 1C). Taken together, these data suggested that mHotair could play a modulatory role during the initial stage of bone development, and that its osteogenic activities could be under the regulation of BMP9.

### Construction and verification of siRNAs targeting murine mHotair in iMADs

**Figure 2:**
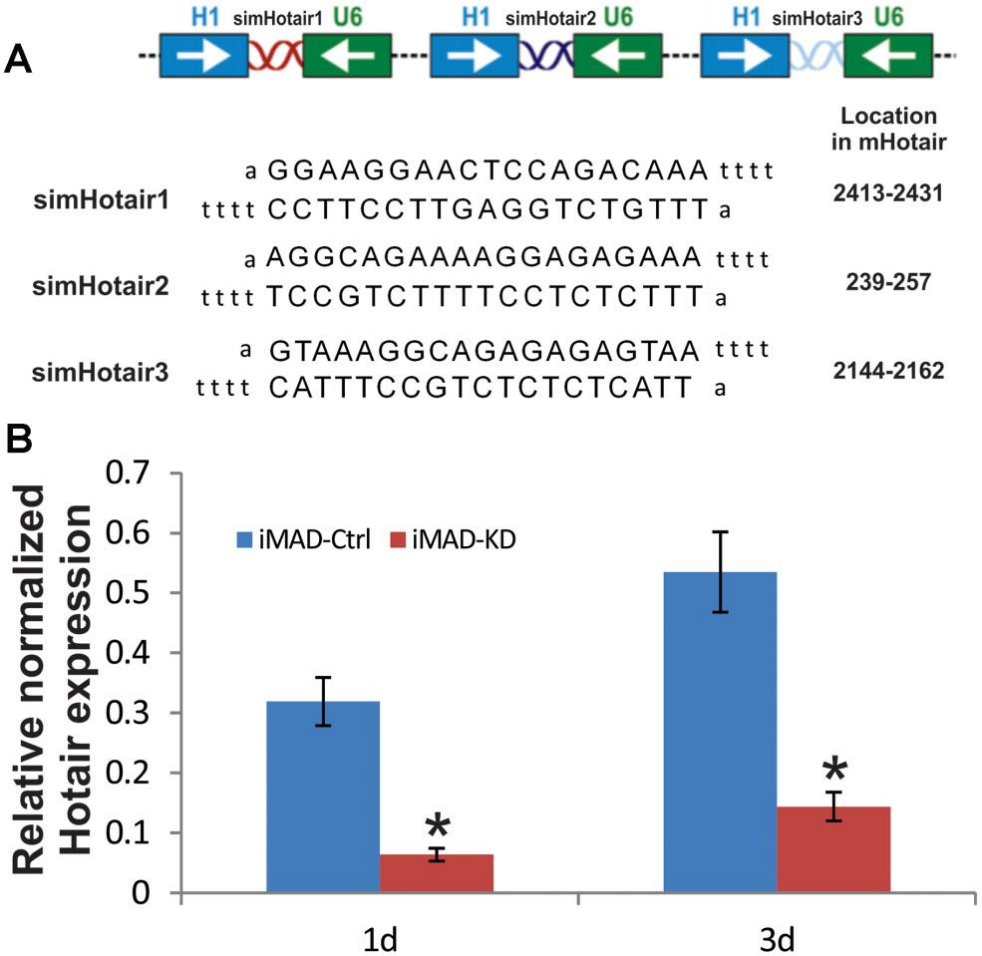
**Construction and verification of siRNA targeting mouse HOTAIR in iMADs.** (**A**) Schematic configuration of the three tandem targeting siRNAs (simHOTAIR1, 2, and 3) and their targeting sequences and locations on mouse HOTAIR gene. (**B**) Efficient silencing of endogenous mHOTAIR expression in iMADs. Total RNA was isolated from subconfluent iMADs that were stably transduced with the pSOK vector expressing three simHOTAIR sites (iMAD-KD) or scrambled controls (iMAD-Ctrl) at Day 1 and 3 post transduction, and subjected to TqPCR analysis using gene-specific primers for mouse HOTAIR. Gapdh was used as a reference gene. Reactions were done in triplicate. “*” p < 0.05, iMAD-Ctrl group vs iMAD-KD group.

We have previously demonstrated our pSOK system to be an effective tool for simultaneous expression of multiple siRNA target sites [28]. Three siRNA sites (Figure 2A), each driven by the opposing U6 and H1 promoters, were subcloned into a modified pSOK vector to stably and effectively silence mHotair expression in iMAD cells, yielding pSOK-simHotair. A control vector in which the siRNAs were replaced with scrambled sequences was also constructed and designated as pSOK-Ctrl. Stable cell lines iMAD-KD and iMAD-Ctrl were obtained by transfecting subconfluent iMAD cells with the above vectors and selected against blasticidin S. TqPCR analysis on day 1 and day 3 of cell culture indicated significant attenuation of mHotair expression in the iMAD-KD group compared to the iMAD-Ctrl group (Figure 2B), confirming that the lncRNA was effectively silenced.

### Silencing mHotair expression significantly diminishes BMP9-induced osteogenic differentiation of iMAD cells *in vitro*

**Figure 3:**
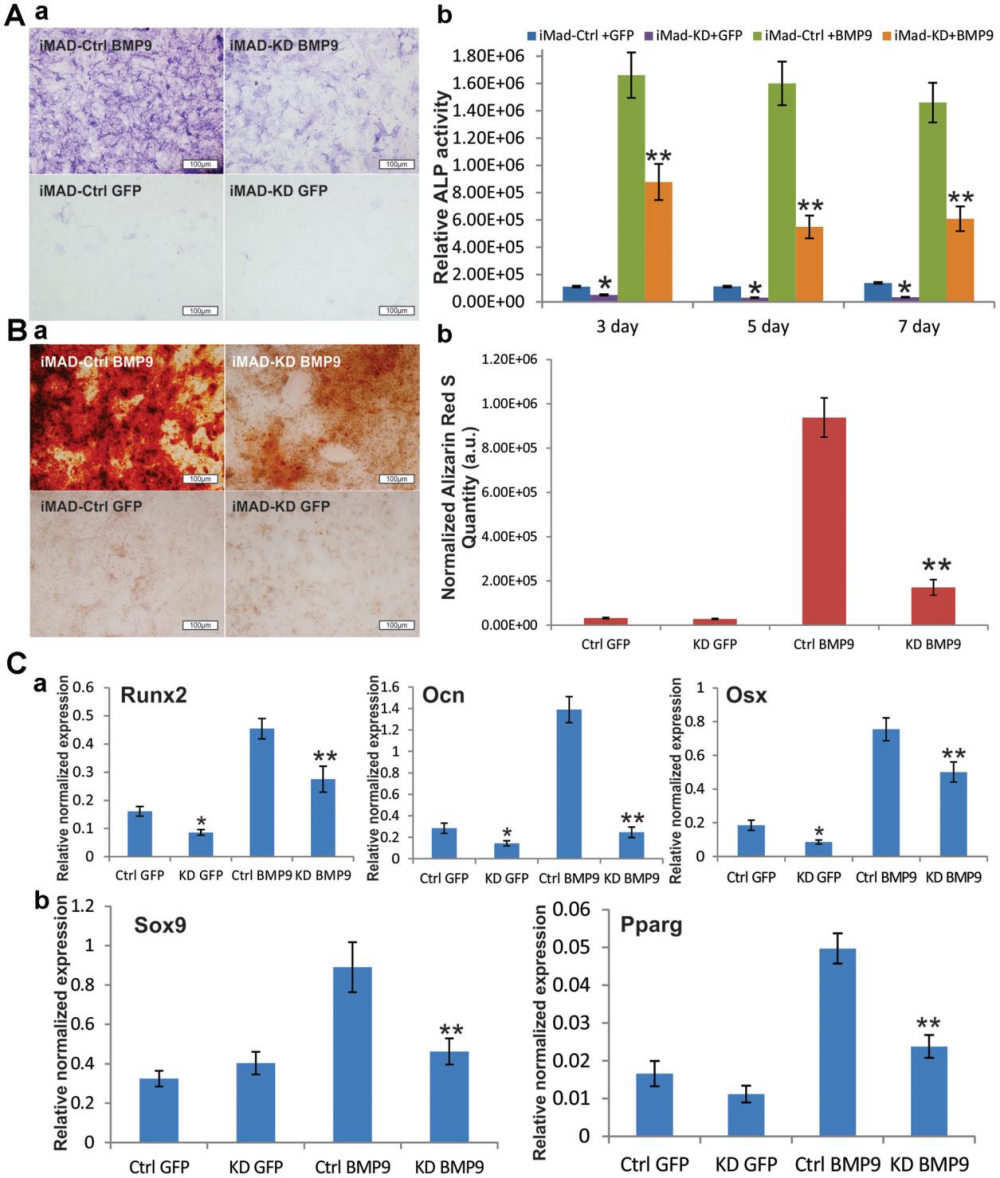
**Silencing HOTAIR expression significantly diminishes BMP9-induced osteogenic differentiation of iMAD cells *in vitro*.** (**A**) Silencing of HOTAIR impairs the endogenous and BMP9-induced activity of ALP in iMADs. Subconfluent iMAD-Ctrl and iMAD-KD cells were infected with Ad-GFP or Ad-BMP9. Qualitative histochemical staining (**a**) and quantitative bioluminescence assay (**b**) were done at days 3, 5, and 7 after infection. (**B**) HOTAIR knockdown significantly diminishes BMP9-induced mineral deposition of iMADs. Subconfluent iMAD-Ctrl and iMAD-KD cells were infected with Ad-GFP or Ad-BMP9 and cultured in mineralization medium for 12 days, and stained with alizarin red S (**a**). Alizarin red S quantification (**b**) was done using the ImageJ program. (**C**) Silencing of HOTAIR down-regulates the endogenous and BMP9-induced expression of osteogenic differentiation markers (**a**), and also diminishes BMP9-induced chondrogenic and adipogenic differentiation markers in iMADs (**b**). Subconfluent iMAD-Ctrl and iMAD-KD cells were infected with Ad-GFP or Ad-BMP9. Total RNA was isolated at 48 h and subjected to TqPCR analysis using gene-specific primers for mouse Runx2, Ocn, Osx, Sox9 and Ppar-γ. All assays were done in triplicate. Representative images are shown. “*” p < 0.05, iMAD-Ctrl+GFP group vs iMAD-KD+GFP group. “**” p < 0.05, iMAD-Ctrl+BMP9 group vs iMAD-KD+BMP9 group.

After iMAD-KD and iMAD-Ctrl were separately infected with BMP9 or GFP, qualitative and quantitative alkaline phosphatase (ALP) assays revealed that BMP9 induced robust ALP activity of the iMAD cells. However, the ALP activity of the iMAD-KD cells was remarkably reduced, regardless of whether BMP9 treatment was involved (Figure 3A), implying that silencing of mHotair could potentially impair the endogenous and BMP9-induced expression of ALP. Alizarin Red S staining further confirmed that BMP9 stimulated significant matrix mineralization in iMAD-Ctrl cells but not so much in iMAD-KD cells (Figure 3B). On the other hand, we have previously demonstrated that BMP9 could drive iMADs into osteoblastic, chondrogenic, and adipogenic lineages. In this regard, TqPCR assays revealed that, in consistence with the results of ALP assays and Alizarin Red S staining, the expressions of osteogenic markers Runx2, Ocn, and Osx in iMAD-Ctrl cells were significantly induced by BMP9, while their expressions in iMAD-KD cells were much lower, even in the GFP-treated groups (Figure 3Ca). On the other hand, although GFP-treated iMAD-KD and iMAD-Ctrl cells showed similar mRNA levels of the chondrogenic marker Sox9 and adipogenic marker Pparg, upon BMP9 induction both genes were significantly down-regulated in the iMAD-KD group compared with iMAD-Ctrl group (Figure 3Cb). Collectively, these results offered convincing evidence that silencing the expression of mHotair could significantly diminish BMP9-dependent osteogenesis, while simultaneously attenuating the chondrogenic and adipogenic differentiation of iMAD cells.

### BMP9-induced ectopic bone formation from iMAD cells can be attenuated by mHotair knockdown

**Figure 4:**
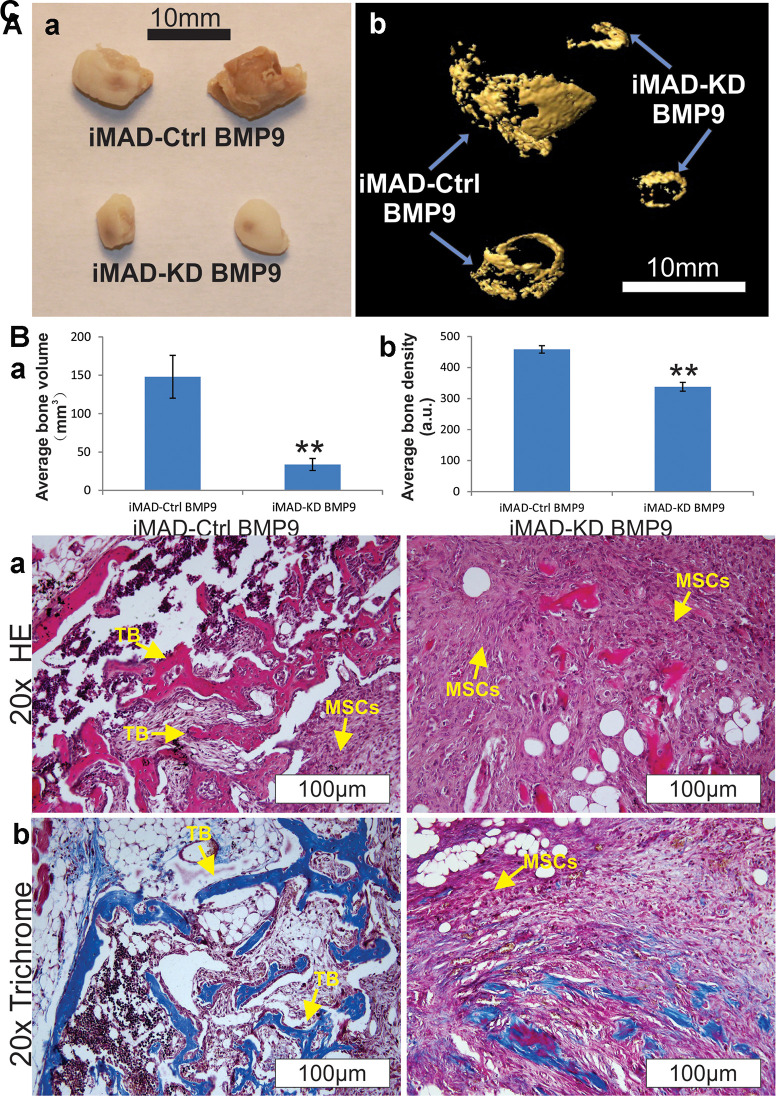
**HOTAIR knockdown attenuates BMP9-induced ectopic bone formation from iMAD cells *in vivo*.** (**A**) Gross view (**a**) and μCT imaging (**b**) of the BMP9-induced ectopic bone formation from iMAD-Ctrl and iMAD-KD cells. The retrieved bone masses from indicated groups were imaged using μCT followed by 3D reconstruction (**b**). No detectable masses were retrieved from the Ad-GFP-transduced iMAD-Ctrl or iMAD-KD cells group. (**B**) The average bone volumes (**a**) and average bone density (**b**) for the indicated groups were determined and analyzed using the Amira program. Representative images are shown. “**” p < 0.05, iMAD-Ctrl+BMP9 group vs iMAD-KD+BMP9 group. (**C**) HE (**a**) and Trichrome staining (**b**) of the retrieved bone masses. Representative images are shown. TB, trabecular bone; MSCs, undifferentiated MSCs.

Cell implantation experiments were conducted to further verify our *in vitro* results. Both iMAD-Ctrl and iMAD-KD cells were infected with BMP9 or GFP and subcutaneously injected into nude mice. Four weeks later, the combination of microCT imaging, H&E and trichrome staining revealed that both the implantation of BMP9-induced iMAD-Ctrl and of iMAD-KD cells, but not either of the GFP-treated groups, led to robust ectopic bone formation. It should be emphasized that BMP9-transduced iMAD-Ctrl cells formed larger and denser bone masses than BMP9-transduced iMAD-KD cells (Figure 4A, 4B). Moreover, histological evaluation detected more trabecular bones and less undifferentiated MSCs in the iMAD-Ctrl/BMP9 group compared with the iMAD-KD/BMP9 group (Figure 4Ca). Trichrome staining indicated that the iMAD-Ctrl/BMP9 group exhibited more extensive mineralization than the iMAD-KD/BMP9 group (Figure 4Cb). Taken together, these results are consistent with the *in vitro* results that we previously reported, and lent strong credence that silencing of mHotair could mitigate BMP9-induced osteogenic differentiation and mineralization of iMAD cells.

### mHotair knockdown significantly enhances the proliferative capabilities of iMAD cells

**Figure 5:**
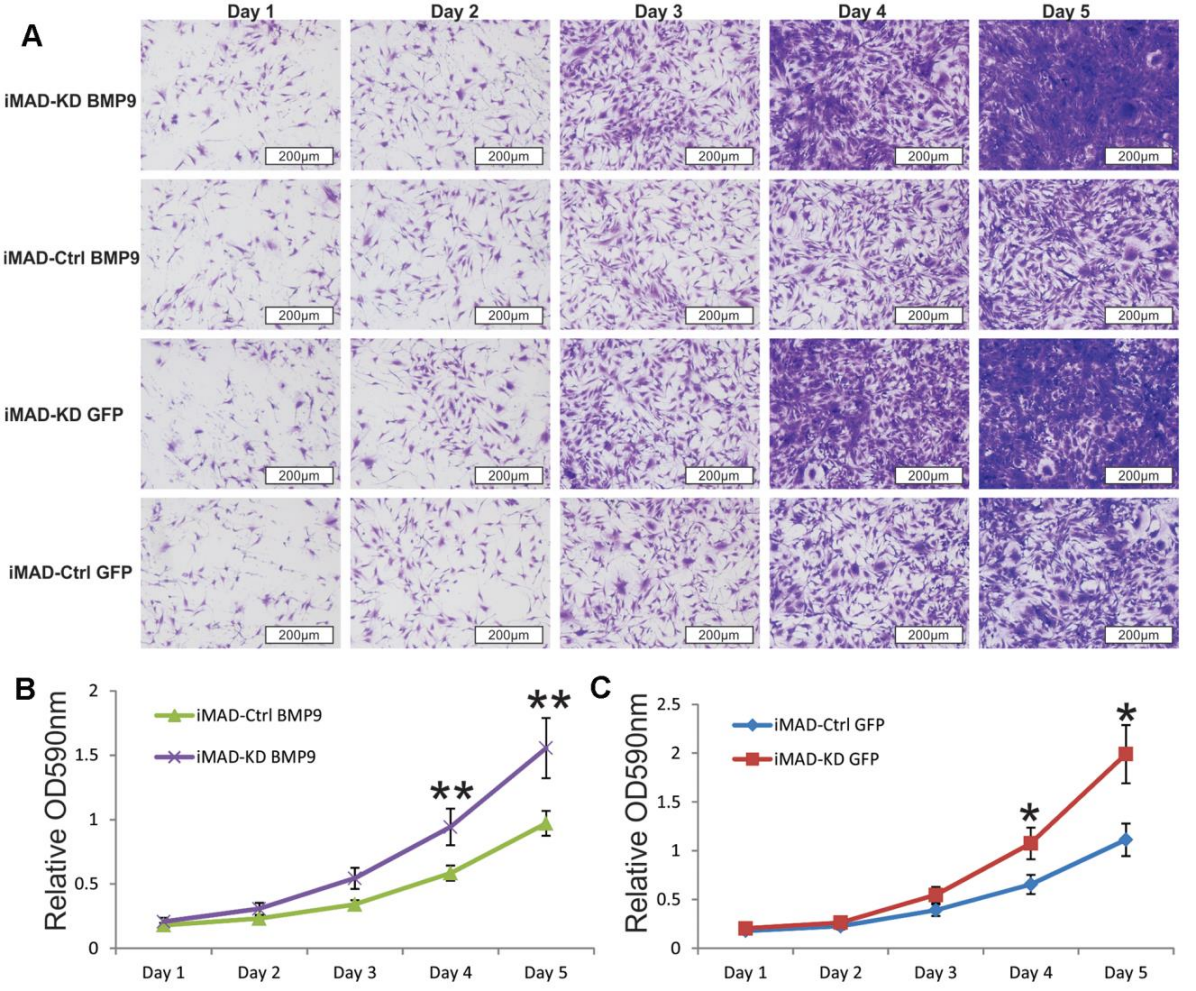
**Silencing HOTAIR expression promotes the proliferative capability of iMAD cells.** (**A**) Subconfluent iMAD-KD and iMAD-Ctrl were infected with Ad-BMP9 or Ad-GFP and fixed for Crystal violet staining at indicated time points. (**B**, **C**) The stained cells were dissolved and quantitatively determined at A590nm. Representative images are shown. “*” p < 0.05, iMAD-Ctrl+GFP group vs iMAD-KD+GFP group. “**” p < 0.05, iMAD-Ctrl+BMP9 group vs iMAD-KD+BMP9 group.

**Figure 6:**
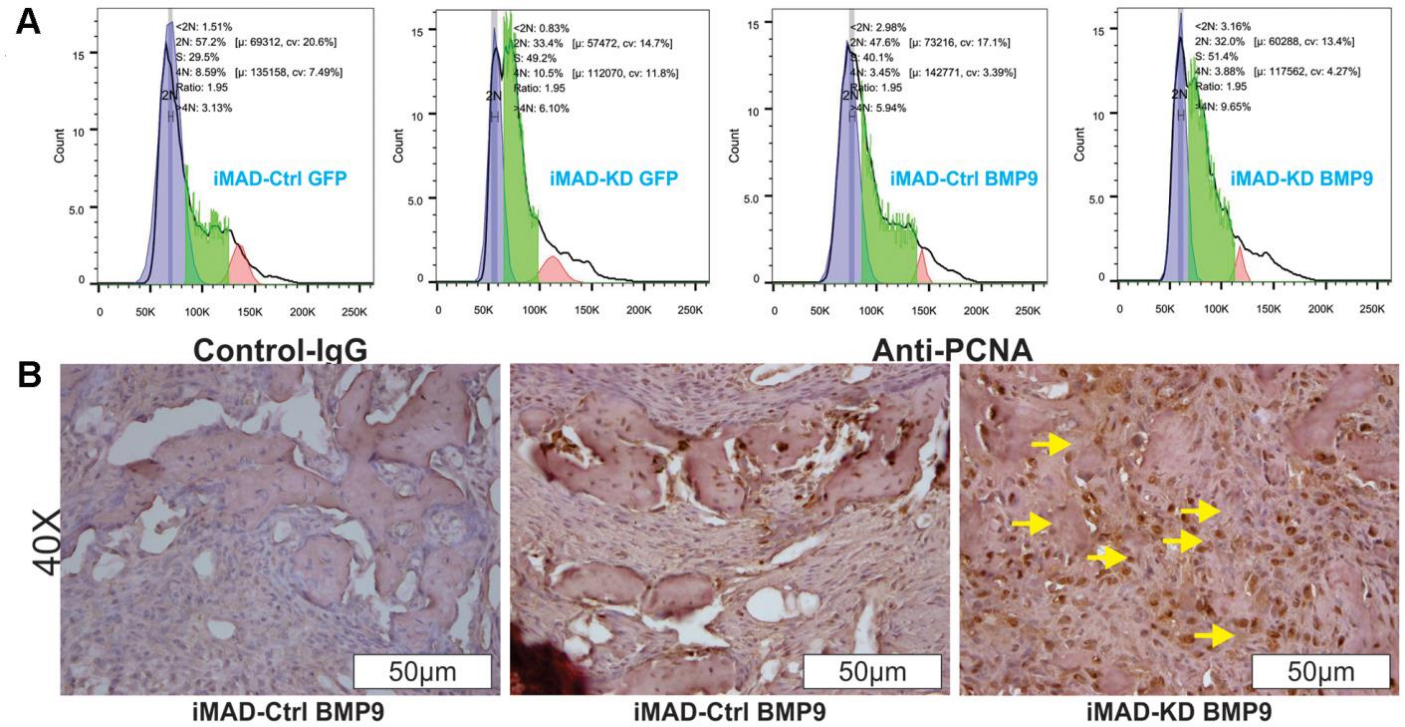
**HOTAIR knockdown switches cell cycle and up-regulates PCNA expression of iMAD cells.** (**A**) Cell cycle analysis. Subconfluent iMAD-KD and iMAD-Ctrl were infected with Ad-BMP9 or Ad-GFP. At 36 h after infection, cells were collected, fixed, stained with Hoechst 33342, and subjected to FACS analysis. Assays were done in triplicate, and representative results are shown. (**B**) Immunohistochemical (IHC) expression analysis of cell proliferation marker PCNA. The retrieved bone masses were sectioned and subjected to IHC staining with either a negative control IgG or PCNA antibody. Representative results are shown. Yellow arrows show positive staining cells.

Since much larger number of undifferentiated MSCs were observed in the iMAD-KD/BMP9 group than in the iMAD-Ctrl/BMP9 group, we hypothesized that the silencing of mHotair could potentiate the proliferative capabilities of iMAD cells. Indeed, crystal violet staining found iMAD-KD cells to proliferate much faster than iMAD-Ctrl cells regardless of BMP9 induction (Figure 5A–5C). Cell cycle analysis further confirmed that suppression of mHotair significantly accelerated G1 phase but lengthened S and S/G2 phases in iMAD-KD cells (Figure 6A). Consistently, immunohistochemical staining of sections prepared from the ectopic bone masses detected significantly higher expression of the cell proliferative marker PCNA in iMAD-KD cells than iMAD-Ctrl cells upon BMP9 induction (Figure 6B). Combined, these findings demonstrated that knockdown of mHotair could significantly promote the proliferation of iMAD cells.

## DISCUSSION

First identified in 2007, HOTAIR has been shown to be implicated in oncogenic progression and tumor metastasis [29–33]. However, literature report of its role in bone development and MSC osteogenesis is rather limited. In this study, we reported that murine mHotair plays important roles in BMP9-induced osteogenesis of MSCs through regulating both cell differentiation and proliferation.

LncRNAs are mostly expressed in a tissue-specific manner. Human Hotair has been shown to be specifically expressed in posterior and distal sites of human body, and murine mHotair is expressed in parts of the proximal hindlimbs, in the posterior part of the body and in the emerging presumptive external genital organs during embryo development. In our study, we found that mHotair was most abundantly expressed in bone tissues. Li also confirmed that knockout of mHotair led to transformation of the spine [27]. Furthermore, our experimental data indicated that peak femoral expression of mHotair coincided with rapid skeletal development in mice. Together, these results suggested that mHotair could play important roles in bone development.

BMP9 belongs to the TGF-beta superfamily and is considered to be one of the most effective osteostimulators. There is ample evidence that BMP9-dependent MSC osteogenesis is subjected to tight regulation by molecular factors such as Notch, wnt3a, and Nell-1 [34–37]. The established role of HOTAIR in bone development thus prompted us to determine whether it could also participate in BMP9 signaling. To our gratification, we found that BMP9 stimulation could expedite peak expression of mHotair. Moreover, silencing of mHotair could promote cell proliferation and inhibit BMP9-induced differentiation of MSCs both *in vivo* and *in vitro*.

HOTAIR has been previously demonstrated to act as a scaffold that couples PRC2 and LSD1, the complex of which functions as a gene repressor by inducing H3K27-trimethylation [33]. Despite its inhibitory role, HOTAIR could also promote the expression of certain genes, including some osteogenic genes [38]. In the current study, our experimental results revealed that silencing of mHotair diminished the expression of osteogenic markers Runx2, Ocn and Osx, chondrogenic marker Sox9, and adipogenic marker Pparg. It is possible that mHotair could directly regulate the expression of these differentiation-associating genes in an epigenetic manner. On the other hand, cytoplasmic HOTAIR might function as a competing endogenous RNA (ceRNA) to modulate the expression of genes involved in cell differentiation and proliferation [30]. It has been reported that HOTAIR could regulate the expression of Notch3 by acting as ceRNA to sponge miR-613 in the context of pancreatic cancer [39]. Thus, it is possible that mHotair could act in a similar manner to regulate BMP9-induced osteogenic differentiation of MSCs.

HOTAIR has also been implicated in ubiquitin-dependent protein degradation, as exemplified by its association with E3 ubiquitin ligases bearing RNA-binding domains such as Dzip3 and Mex3b [40]. The finding that Mex3b could help HOTAIR interact with Smad-4 [41, 42], a key component of BMP signaling, strongly implies the role of HOTAIR in the regulation of TGF-beta superfamily signaling.

Interestingly, a recent studies reported that the late-stage osteogenic marker osteopontin (OPN), which is an extracellular matrix protein secreted by osteoblasts and osteocytes, can transcriptionally activate and increase HOTAIR expression in cancer cells [43]. Although the expression of OPN was not examined in this study, silencing of mHotair was shown to inhibit bone generation and down-regulate Runx-2 as well as OCN, which could theoretically down-regulate the expression of OPN and thus lead to further inactivation of mHotair. Thus, it is conceivable that OPN and HOTAIR could form a positive feedback loop in the regulation of MSC osteogenesis.

It should be stressed that murine mHotair lacks sequence homology with its human counterpart HOTAIR despite their functional similarity [44, 45], suggesting that the function of this lncRNA might largely depend on its secondary structure rather than its sequence. The structure of HOTAIR can be divided into four distinct domains designated as D1 to D4, with more than 50% of the nucleotides involved in base pairing. Covariance analysis across different mammalian HOTAIR sequences revealed that Helix-7 in D1, the proposed binding site for PRC-2, is highly conserved. Specifically, mHOTAIR and its human equivalent share 25 identical nucleotides out of 29 that comprise Helix-7. Moreover, conserved helices are present through all four domains of HOTAIR, but not only limited to predicted protein-binding regions. These data demonstrated that a number of common human and mouse HOTAIR elements are evolutionarily preserved [46].

## CONCLUSIONS

To summarize, we found that mHotair levels were generally greater in bones than in most other tissues. Also, peak femoral expression of mHotair coincided with rapid skeletal development in mice. By investigating the role of mHotair in BMP9-induced osteogenic differentiation of MSCs, we found knockdown of mHotair diminished BMP9-induced ALP activity, matrix mineralization, expression of osteogenic markers (Runx2, Ocn and Osx), chondrogenic marker Sox9, and adipogenic marker Pparg *in vitro*. Furthermore, silencing of mHotair attenuated the BMP9-induced ectopic bone formation and mineralization while promoting cell proliferation. Our findings demonstrate mHotair is an important regulator of BMP9-induced osteogenic differentiation of MSCs by targeting cell cycle and proliferation.

## MATERIALS AND METHODS

### Cell culture and chemicals

Briefly, iMAD [7], HEK-293 (from ATCC, Manassas, VA) and 293pTP cells [47] were maintained at 37° C under an atmosphere of 5% CO_2_ in complete Dulbecco’s Modified Eagle Medium (DMEM), supplemented with 10% (v/v) fetal bovine serum (FBS; Invitrogen, Carlsbad, CA, USA), 100 μg/ml streptomycin and 100 U/ml penicillin. All chemicals were purchased from Sigma-Aldrich (St. Louis, MO, USA) or Thermo Fisher Scientific (Waltham, MA, USA) unless indicated otherwise.

### Generation of BMP9-expressing recombinant adenoviruses

The AdEasy Adenoviral Vector System was used for the generation of recombinant adenoviruses [48–50]. The coding sequence of human BMP9 was PCR-amplified and inserted into the commercial adenoviral shuttle vector that also encodes a green fluorescent protein (GFP). Recombinant adenoviruses, designated as Ad-BMP9, were then produced by transfecting HEK-293 or 293pTP cells with the BMP-encoding vector in the presence of 4 – 8 μg/ml polybrene as enhancer [47, 51, 52] GFP-only control viruses, designated as AdGFP, were generated in a similar manner but with the insert-free vector [11, 34].

### Generation of stable cell lines iMAD-KD and iMAD-Ctrl

The sequences encoding three mHotair-targeting siRNA sites were obtained from GenBank, PCR-amplified, and cloned into the retroviral vector pSOK [28], yielding pSOK-simHotair plasmid. A control plasmid, pSOK-Ctrl, was similarly generated with a scrambled sequence. Subconfluent iMAD cells were transfected with pSOK-simHotair or pSOK-Ctrl for 48 h and selected against 4 mg/ml blasticidin S over the next 5-7 days to generate iMAD-KD or iMAD-Ctrl, respectively, as stable lines. The primer sequences used in the PCR are listed in Figure 2A.

### Alkaline phosphatase (ALP) assays

Subconfluent iMAD-Ctrl and iMAD-KD cells were seeded in 24-well culture plates, infected with AdBMP9 or AdGFP, and grown in complete DMEM. The cells were then harvested at the indicated days, and stained with a mixed solution of 0.1 mg/ml napthol AS-MX phosphate and 0.6 mg/ml Fast Blue BB salt. ALP activity was measured by using the Great Escape SEAP Chemiluminescence Assay Kit (BD Clontech, Mountain View, CA, USA) following a previously reported protocol [53, 54].

### Matrix mineralization assay (Alizarin red S staining and quantification)

Subconfluent iMAD-Ctrl and iMAD-KD cells were infected as above in the presence of 50 μg/mL ascorbic acid and 10 mM β-glycerophosphate. The cells were then collected at the indicated days and fixed with 0.05% (v/v) glutaraldehyde for 10 min at room temperature. After rinsing with distilled water to remove the fixative, the cells were stained with 0.4% Alizarin Red S for 5 min, rigorously washed with distilled water, and visualized under a bright-field microscope to evaluate the formation of mineralized calcium nodules [12, 51]. Quantitative analysis of Alizarin Red dye was conducted with ImageJ.

### Total RNA isolation and TqPCR analysis

Total RNA was isolated from both mouse tissues and cell lines with TRIzol (Thermo Fisher Scientific, Waltham, MA, USA). Specifically, various types of tissues were harvested from CD1 male mice at the indicated development stages (n = 5 at each time point), immediately rinsed with PBS, and homogenized in TRIzol. On the other hand, subconfluent iMAD-Ctrl and iMAD-KD cells were inoculated into 60 mm culture dishes, infected with AdGFP or AdBMP9, and treated with TRIzol at the indicated time points. In both cases, cDNA was synthesized from the extracted total RNA using random hexamer and M-MuLV Reverse Transcriptase (New England Biolabs, Ipswich, MA, USA), and subsequently diluted 10- to 50-fold before being used as PCR template. TqPCR analysis of HOTAIR was performed using the following conditions, 95° C × 3 min for one cycle; 95° C × 20 sec, 66° C × 10 sec, for 4 cycles by decreasing 3° C per cycle; 95° C × 20 sec, 55° C × 10 sec, 70° C × 1 sec, followed by plate read, for 40 cycles [55, 56]. *Gapdh* was used as a reference gene. The TqPCR primers are listed in Supplementary Table 1.

### Ectopic bone formation, micro–computed tomographic (μCT) imaging and histological analysis

All animal studies were approved by the Institutional Animal Care and Use Committee and performed in accordance with the established guidelines. A total of 24 female athymic nude mice (Envigo/Harlan Research Laboratories, Indianapolis, IN, USA) of 5 – 6 weeks old were randomly divided into four equal-sized experiment groups, including iMAD-Ctrl + AdBMP9, iMAD-Ctrl + AdGFP, iMAD-KD + AdBMP9, and iMAD-KD + AdGFP, and subjected to subcutaneous stem cell implantation as previously elucidated [12, 28, 57]. The iMAD-Ctrl and iMAD-KD cells were first infected with AdBMP9 or AdGFP for 24 h, pelleted, rinse in PBS, and then injected subcutaneously into female athymic nude mice at a dose of 2 × 10^6^ cells per injection site. The mice were housed under normal conditions with access to food and water ad libitum, and sacrificed at 4 weeks after the cell implantation. The ectopic masses were resected from the injection sites, fixed in 10% (v/v) formalin, and underwent micro-CT (μCT) imaging on a GE Triumph tri-modality preclinical imaging system (GE Healthcare, Chicago, IL, USA). The image data were analyzed by using Amira 5.3 (Visage Imaging, San Diego, CA, USA) to estimate the mass sizes. After μCT imaging, the specimens were decalcified, embedded in paraffin, and cut into 5-μm sections. The sections were deparaffinized and stained with H&E as described [7, 53]. Trabecular bone area was measured using ImageJ.

### Crystal violet assay

Subconfluent iMAD-Ctrl and iMAD-KD cells were inoculated into 35 mm cell culture dishes and infected with Ad-BMP9 or Ad-GFP. At the indicated time points, the infected cells were stained with crystal violet [58, 59] and visualized under a bright field microscope. For quantitative measurement, the stained cells were dissolved with agitation in 10% acetic acid for 20 min at room temperature and the absorbance of the resultant mixture was determined at 570~590 nm based on procedures reported in previously studies [60, 61].

### Fluorescence-activated cell sorting (FACS) analysis

Subconfluent iMAD-Ctrl and iMAD-KD cells were inoculated into 60 mm cell culture dishes and infected with Ad-BMP9 or Ad-GFP for 24 h. Then, the cells were harvested, fixed and stained with Hoechst 33342. Cell cycles were analyzed on a BD LSR II Flow Cytometer and data were processed by FlowJo as previously described [7, 62, 63].

### Immunohistochemical staining for PCNA

IHC staining was conducted according to previously described protocols [61, 64, 65]. Briefly, the sections were deparaffinized, subjected to antigen unmasking, and subsequently immunostained with anti-PCNA antibodies (Santa Cruz Biotechnology, Santa Cruz, CA, USA). Control IgG and minus primary antibodies were used as negative controls.

### Statistical analysis

All quantitative assays were performed in triplicate and/or in three independent batches. For animal studies, sample size was determined by Sample Size Calculator software. Statistical analyses were conducted using Microsoft Excel. Data were expressed as mean ± SD. Differences between experiment groups were analyzed by one-way analysis of variance and the student’s t test. P < 0.05 was considered statistically significant.

## Supplementary Material

Supplementary Table 1
