# Supplementary material for: Long non-coding RNA (LncRNA) HOTAIR regulates BMP9-induced osteogenic differentiation by targeting the proliferation of mesenchymal stem cells (MSCs)
Source: Aging (Albany NY). 2021 Jan 10;13(3):4199–214. doi: 10.18632/aging.202384 (PMC7906180; doi:10.18632/aging.202384)
Supplement: Supplementary Table 1 [file aging-13-202384-s001.pdf]

SUPPLEMENTARY TABLE

Supplementary Table 1. List of qPCR primers.

Genes	Primer sequences	Accession No.
Mouse Runx2	GCCGGGAATGATGAGAACTA	NM_009820
	GGACCGTCCACTGTCACTTT	
Mouse Sox9	GCAAGCAAAGGAGACCAAAA	NM_011448
	CGCTGGTATTCAGGGAGGTA	
Mouse Ppar-γ	TTTTCAAGGGTGCCAGTTTC	NM_011146
	AATCCTTGGCCCTCTGAGAT	
Mouse Osteocalcin(Ocn)	CCTTCATGTCCAAGCAGGA	NM_001032298
	GGCGGTCTTCAAGCCATAC	
Mouse Osterix(Osx)	GAAGTCCAATGGGGATCTGA	NM_130458
	AGAATCCCTTTCCCTCTCCA	
Mouse Gapdh	ACCCAGAAGACTGTGGATGG	NM_008084
	CACATTGGGGGTAGGAACAC	
